# The challenges of a randomised placebo-controlled trial of CTO PCI vs. placebo with optimal medical therapy: The ORBITA-CTO pilot study design and protocol

**DOI:** 10.3389/fcvm.2023.1172763

**Published:** 2023-05-03

**Authors:** Sarosh Khan, Samer Fawaz, Rupert Simpson, Craig Robertson, Paul Kelly, Shah Mohdnazri, Kare Tang, Christopher M. Cook, Sean Gallagher, Peter O’Kane, James Spratt, Emmanouil S. Brilakis, Grigoris V. Karamasis, Rasha Al-Lamee, Thomas R. Keeble, John R. Davies

**Affiliations:** ^1^ Department of Interventional Cardiology, Essex Cardiothoracic Centre, Basildon, United Kingdom; ^2^ Department of Interventional Cardiology, Anglia Ruskin University, Chelmsford, United Kingdom; ^3^ Department of Interventional Cardiology, University Hospital of Wales, Cardiff, United Kingdom; ^4^ Department of Interventional Cardiology, Royal Bournemouth Hospital, Bournemouth, United Kingdom; ^5^ Department of Interventional Cardiology, St. George's Hospital, London, United Kingdom; ^6^ Minneapolis Heart Institute and Minneapolis Heart Institute Foundation, Abbott Northwestern Hospital, Minneapolis, United States; ^7^ School of Medicine, Attikon University Hospital, National and Kapodistrian University of Athens, Athens, Greece; ^8^ National Heart and Lung Institute, Imperial College London, London, United Kingdom

**Keywords:** chronic total occlusion (CTO), percutaneous coronary intervention (CTO), chronic coronary artery disease, chronic coronary syndrome (CCS), stable angina

## Abstract

**Background:**

Percutaneous coronary intervention (PCI) for coronary chronic total occlusion (CTO) has been performed for the improvement of symptoms and quality of life in patients with stable angina. The ORBITA study demonstrated the role of the placebo effect in contemporary PCI in non-CTO chronic coronary syndromes. However, the benefit of CTO PCI beyond that of a placebo has not been demonstrated.

**Aims:**

The ORBITA-CTO pilot study will be a double-blind, placebo-controlled study of CTO PCI randomising patients who have: (1) been accepted by a CTO operator for PCI; (2) experienced symptoms due to a CTO; (3) evidence of ischaemia; (4) evidence of viability within the CTO territory; and (5) a J-CTO score ≤3.

**Methods:**

Patients will undergo medication optimisation that will ensure they are on at least a minimum amount of anti-anginals and complete questionnaires. Patients will record their symptoms on an app daily throughout the study. Patients will undergo randomisation procedures, including an overnight stay, and be discharged the following day. All anti-anginals will be stopped after randomisation and re-initiated on a patient-led basis during the 6-month follow-up period. At follow-up, patients will undergo repeat questionnaires and unblinding, with a further 2-week unblinded follow-up.

**Results:**

The co-primary outcomes are feasibility (blinding) in this cohort and angina symptom score using an ordinal clinical outcome scale for angina. Secondary outcomes include changes in quality-of-life measures, Seattle Angina Questionnaire (SAQ), peak VO2, and anaerobic threshold on the cardiopulmonary exercise test.

**Conclusion:**

The feasibility of a placebo-controlled CTO PCI study will lead to future studies assessing efficacy. The impact of CTO PCI on angina measured using a novel daily symptom app may provide improved fidelity in assessing symptoms in patients with CTO's.

## Introduction

Chronic total occlusions (CTOs) are present in 18%–50% of patients with coronary artery disease undergoing diagnostic angiography (1–3). Percutaneous coronary intervention (PCI) is increasingly being performed globally due to a combination of improved technique, experience, and equipment (4, 5). Recent randomised controlled trials did not demonstrate a reduction in the incidence of major adverse cardiac events with CTO PCI but were underpowered (6, 7). Therefore, the primary indication for CTO PCI remains symptom relief (8) which is based on large cohorts and unblinded randomised data (6, 9, 10). The benefit of CTO PCI beyond that of placebo in a blinded trial has not been demonstrated. After the Objective Randomised Blinded Investigation with optimal medical Therapy of Angioplasty in stable angina (ORBITA) (11) study established the role of the placebo effect in contemporary PCI for non-CTO chronic coronary syndrome patients, the need for evaluation of CTO PCI's benefit beyond that of placebo is paramount.

The SHINE-CTO (Sham-Controlled Intervention to Improve QOL in CTOs) randomised controlled trial was designed to address this need; however, the study was terminated at a very early stage due to funding issues during the COVID-19 pandemic (ClinicalTrials.gov: NCT02784418). All placebo-controlled studies are difficult to conduct given the challenge of achieving robust blinding; however, a placebo-controlled study of CTO PCI has multiple difficulties beyond those of a conventional PCI trial. Accordingly, this significantly increases the complexity of such a study. With this in mind, we designed and described a randomised placebo-controlled pilot trial of CTO PCI vs. placebo with optimal medical therapy (ORBITA-CTO Pilot) to establish the feasibility of placebo-controlled study in the challenging CTO population. In this manuscript, we discuss the challenges of such a study and focus on the methodology adopted to optimise blinding and maximise trial integrity.

## Methods and analysis

### Design

**Figure 1:**
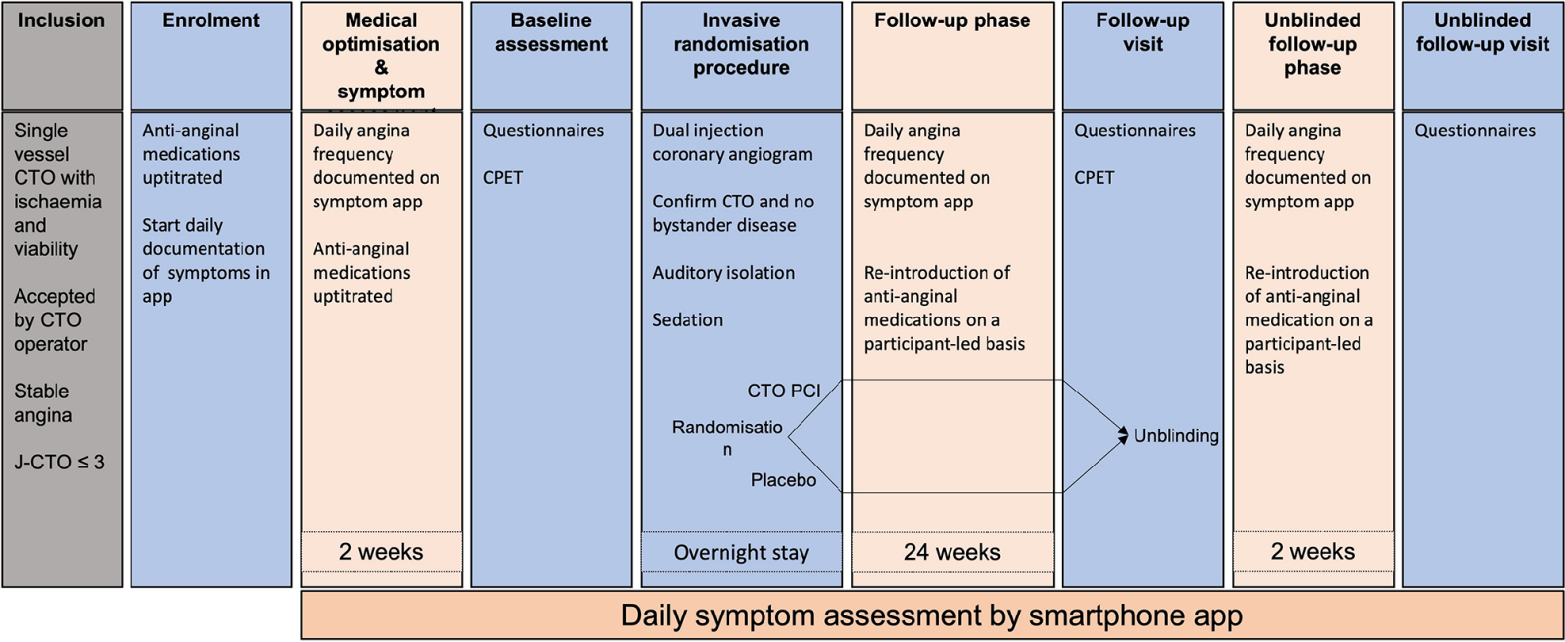
ORBITA-CTO Pilot study design. CPET: cardiopulmonary exercise test; CTO: chronic total occlusion; J-CTO: Japan chronic total occlusion score; PCI: percutaneous coronary intervention.

ORBITA-CTO Pilot (ClinicalTrials.gov Identifier: NCT05142215) is a double-blind, randomised, placebo-controlled trial conducted in the United Kingdom to assess the feasibility of a placebo-controlled study in CTO PCI. It will provide data that will be used to power a larger study to test the efficacy of CTO PCI compared with placebo on angina in patients with background medical therapy. The study design is shown in Figure 1.

### Ethics

The Bradford Leeds Research Ethics Committee (21/YH/0165), United Kingdom, approved the study.

### Eligibility

The inclusion criteria are shown in Table 1. ORBITA-CTO Pilot will enrol participants who are symptomatic due to a single-vessel CTO and who are deemed eligible for CTO PCI at a dedicated multi-disciplinary team (MDT) meeting. Myocardial ischaemia and viability must be demonstrated in the CTO territory by either myocardial nuclear perfusion, cardiac magnetic resonance imaging (with perfusion and gadolinium enhancement) or dobutamine stress echocardiography. The CTO vessel must be at least 2.5 mm in diameter, have a Japan-Chronic Total Occlusion (J-CTO) score of ≤3, with no significant coronary artery stenosis in remaining non-CTO vessels (diameter ≥2 mm) defined as: left main stem ≥50%, left anterior descending, /circumflex/right coronary artery/bypass graft ≥70%. The absence of clinically significant bystander disease is important to avoid the pitfalls of assessing multi-vessel PCI (7). Detailed exclusion criteria are available in Appendix A.

**Table 1:** Inclusion Criteria.

Inclusion Criteria
1. Accepted for CTO PCI procedure by a specialist CTO operator.
2. Patients with symptoms related to a single vessel CTO (≥3 months duration, or probable CTO where duration is unknown) in a vessel of at least 2.5mm diameter without angiographically significant (LMS ≥50%, LAD/Cx/RCA/Graft ≥70%, ≥2mm diameter) coronary artery stenosis in remaining non-CTO vessels. Symptoms are: a) Typical exertional angina defined as: i) constricting discomfort in the front of the chest or the neck, jaw, shoulder or arm ii) precipitated by physical exertion iii) relieved by rest or nitrates within 5 minutes b) Angina symptoms at rest (including decubitus angina and post-prandial angina). c) Shortness of breath on exertion considered to be angina equivalent.
3. Clinical evidence of ischaemia in CTO territory on dobutamine stress echocardiography, nuclear myocardial perfusion scan, stress perfusion cardiovascular magnetic resonance (CMR) or positron emission tomography (PET). 4. Evidence of viability: If left ventricular angiogram or echocardiogram demonstrates left ventricular (LV) impairment or regional wall motion abnormality (RWMA) then viability must be demonstrated.
5. J-CTO score ≤3.

#### Rationale for inclusion and exclusion criteria

This study aims to replicate clinical practice while at the same time ensuring a robust research methodology. Therefore, symptoms will be assessed using the ORBITA-2 online daily angina symptom application, allowing a tailored assessment of each participant's symptoms (12). If participants have symptoms that are their angina, then this would, as in clinical practice, be sufficient to consider them symptomatic. Patients must remain symptomatic after medical optimisation and be symptomatic in the week prior to randomisation. Data from the angina symptom app will be analysed using the ordinal clinical outcome scale, meaning that severity of angina, anti-anginal medication use, and clinical events are incorporated into the primary efficacy endpoint.

The ISCHEMIA-CTO trial utilises an ischaemic burden of 5% for inclusion, combined with the results of the ISCHAEMIA trial (13), this demonstrates the ambiguity of the relationship between ischaemia and symptoms. Acknowledging this discrepancy, we have not included an ischaemic threshold within our study, instead opting for a more pragmatic and clinical approach. Furthermore, although it is ideal to have a single modality for ischaemia and viability assessment, within clinical practise this would not be practical and therefore was not mandated as a research procedure.

The J-CTO score predicts the probability of a wire crossing a CTO within 30 min. It is not a tool used for patient selection but rather to assess the complexity of CTO lesion. The higher the J-CTO score, the higher the probability of technical failure (14). A large proportion of CTO's intervened upon in Europe have a J-CTO score between 0 and 3 (15). Therefore, this parameter was chosen to avoid a high, predictable CTO-PCI procedural failure rate while maintaining acceptable external validity. The screening record will include participants who were not enrolled due to a J-CTO 4 or 5 score.

## Outcomes

### Primary outcome

Fidelity of blinding using Bang's blinding index (16).

Protocol adherence.

### Primary efficacy outcome

Change in daily angina ordinal clinical outcome scale.

### Secondary outcomes

Change in Seattle Angina Questionnaire (SAQ) physical limitation, angina frequency, angina stability, treatment satisfaction, and quality of life scores.
Change in SAQ summary score.
Quality of life as measured by EQ-5D-5L.
Change in Rose dyspnoea scale.
Change in peak VO2 and at anaerobic threshold (AT).
Change in cardiopulmonary exercise testing (CPET) parameters.
Cost of treatment.

### Enrolment

Patients identified at the CTO multi-disciplinary team meeting who meet the eligibility criteria will be approached by the CTO consultant for CTO PCI and offered participation in the research study. Patients indicating a willingness to participate will be approached by the research team and provided with written documentation about the research at least 48 h before enrolment.

At enrolment, written informed consent will be obtained. Participants' angina symptoms will be assessed. Participants will be taught how to use the smartphone symptom application for recording their daily symptoms. The smartphone application notifies the research team if participants have failed to report symptoms. If 3 days or more are missed, participants will be prompted by research staff to enter their symptoms.

#### Pre-randomisation assessment

All participants will have their symptoms assessed according to the Canadian Classification for Angina (CCS), NYHA, and Rose dyspnoea scale. Participants will complete an SAQ and EQ-5D-5L for quality-of-life assessments. All participants will undergo a physical examination, observations, and an electrocardiogram.

#### Medication optimisation

Following enrolment, participants will have protocolised anti-anginal medication optimisation as previously described in the ORBITA-2 study (12).

#### Anti-anginal medication

Anti-anginal medications will be optimised on enrolment and additional uptitration will be undertaken only at the instruction of the research team. An individualised protocol for the potential introduction of anti-anginal medications will be prepared by the research team in conjunction with the participant at enrolment. This protocol will be based on their medical history, heart rate, blood pressure, and any intolerances. See Appendix B for the sequence of introduction of anti-anginals and their units.

The aim will be to establish participants on at least 3 units of anti-anginal medications (e.g., Bisoprolol 10 mg OD and Amlodipine 2.5 mg OD) for at least 1 week before undergoing baseline assessments. Participants who are already taking 3 units or more of anti-anginals at the time of enrolment will not require any changes to their anti-anginal medications. After participants are established on 3 units of anti-anginals for 48 h, they will be considered medically optimised. Participant symptoms will then be monitored for 1 week prior to randomisation to ensure they remain symptomatic while medically optimised. If participants become asymptomatic, therefore no angina during the 1-week period after medical optimisation, they will exit the study, and their usual medical care will continue.

Participants will stop all anti-anginals medication after randomisation. Anti-anginals will subsequently be re-introduced, as per their individualised protocol established at enrolment, in the blinded follow-up period by participant-led interactions with the blinded study team.

### Pre-randomisation assessments

Prior to the randomisation procedure, participants will undergo a repeat assessment, including the CCS class, the NYHA, the Rose dyspnoea assessment of symptoms, and the completion of the SAQ and EQ-5D-5L. Participants will have CPET performed unless they are physically unable.

### Randomisation invasive procedure and overnight hospital stay

Prior to the procedure, all participants will have consented for coronary angiography +/− CTO PCI. Participants will not be told the expected duration of the procedure to reduce the chance of inadvertent unblinding. Participants will be prepared for PCI as per normal clinical practice.

Double arterial access and dual-injection coronary angiography will be performed. A minimum number of angiogram images will be used, to confirm both CTO presence and absence of bystander disease, to reduce contrast load and radiation exposure.

Incremental boluses of IV benzodiazepines and IV opiates will be administered to achieve and maintain a deep level of conscious sedation such that participants are unresponsive to voice and tactile stimuli with no need for airway, ventilatory, or haemodynamic support.

Participants will then be randomised 1:1 to CTO PCI or placebo procedure using a computerised online randomisation tool. Randomisation will be performed by an unblinded member of the research team immediately after the performance of the dual-injection coronary angiography, confirming the CTO and excluding bystander disease. If the CTO has recanalised or the patient has significant bystander disease, then the patient will be withdrawn from the study, and PCI to the recanalised vessel or bystander disease will proceed. Patients are aware of this prior to signing the research consent form and also on the day of the procedure when signing the routine clinical procedural consent form.

Those randomised to CTO PCI will have their procedure performed by CTO specialist operators using contemporary techniques, algorithms, and equipment. Further intra-arterial or IV heparin will be administered as per contemporary practice. Recanalisation strategy will be at the operator's discretion. Intravascular imaging, e.g., intravascular ultrasound, will be used to guide the procedure and for stent planning and optimisation as per contemporary interventional practice. A procedure will be considered successful when it achieves thrombolysis in myocardial infarction (TIMI) flow grade 2–3 with <30% angiographic residual stenosis in the CTO vessel (17). All participants will receive drug-eluting stents. A subgroup of participants in the intervention arm will undergo invasive coronary physiological assessment after the completion of CTO PCI.

Participants randomised to placebo, will have diagnostic catheters removed, leaving only bilateral arterial sheaths *in-situ*. No further contrast or radiation will be used to minimise risk. A deep level of conscious sedation will be maintained for a total procedural time of 60 minutes after randomisation.

All participants will remain overnight in a cardiology ward post-procedure. Participants will be discharged on dual antiplatelet therapy with duration of at least 6 months, according to expert guidance. All anti-anginal medication will be stopped. The discharge summary to the General Practitioner (GP) will state that unblinding will occur after 24 weeks and that all participant anti-anginals have been stopped. Anti-anginal medications will only be re-introduced on a participant-led basis if they experience recurrent symptoms after consultation with the blinded research fellow (SK) and blinded research consultant (RAL). Any patients requiring the continuation of medications that have anti-anginal properties but are required for another indication e.g., Bisoprolol for congestive cardiac failure, that do not have an alternative substitute agent without anti-anginal properties, will be continued and documented as an anti-anginal uptitration and reflected as such in the ordinal clinical outcome scale.

### Randomisation and blinding

Participants will be randomised 1:1 to either the PCI or placebo procedure using computer-generated randomisation with block randomisation using Randi (open-source clinical trial software). Randomisation will be performed in the catheterisation laboratory. The allocation will be communicated verbally to the catheterisation laboratory team and the unblinded research study team. Prior to discharge, the fidelity of blinding will be assessed using the blinding index (16) for participants, the blinded research member, and the ward team.

### Follow-up evaluation

At the 6-month (24 weeks) follow-up visit, the fidelity of blinding at follow-up will be assessed using the blinding index for participants and the research team before follow-up assessments are performed. Participants will then be assessed using the CCS, NYHA, Rose dyspnoea scale, EQ-5D-5L and the SAQ. Participants will also undergo a repeat CPET assessment. Participants will then be unblinded.

### Unblinded follow-up

All participants will enter the unblinded 2-week follow-up phase after the follow-up evaluation. During this period, they will continue to complete the daily smartphone symptom application and have re-introduction of anti-anginals on a participant-led basis. At 2 weeks, they will have a repeat questionnaire assessment of symptoms. This period will assess the impact of unblinding on participant symptoms. The data collected during this period will not contribute to the primary and secondary outcomes.

### Study completion

Study participation will be complete after the unblinded follow-up visit. Participants in the placebo arm will be offered CTO PCI if clinically indicated after consultation. Participants will return to routine clinical care.

### Sample size

Assessing the feasibility of the first double-blind, placebo-controlled CTO PCI trial necessitated the need for a pilot study. Should feasibility be demonstrated in this pragmatic, 50 patient pilot study, the efficacy data will be used to calculate the sample size for a larger pivotal study.

### Statistical analysis plan

Data will be summarised as quartiles for continuous variables and proportions for categorical ones. The data will be analysed on an intention-to-treat basis. The primary outcome of the ORBITA-CTO Pilot is the placebo-controlled efficacy of PCI on the angina symptom score using an ordinal clinical outcome scale for angina. The primary outcome will be analysed using the same methodology as in the ORBITA-2 (12) study, with a comparison of the ordinal clinical outcome scale between groups. The statistical packages SPSS and R will be used for computations.

### Safety and monitoring

A data safety monitoring board (DSMB) will be established, consisting of clinicians and researchers who are not participating in the trial but have knowledge of trial management and insight into the subject matter. The DSMB will meet regularly throughout the trial to review and adjudicate all events, including the re-introduction of anti-anginal medications during follow-up, and report to all relevant regulatory bodies. Recommendations to any modifications to protocol, consent, documentation, or advisory to continue study unchanged will be provided upon conclusion of meetings.

### Dissemination plan

After completion of primary and secondary analyses, ORBITA-CTO Pilot will be presented at a cardiology conference and published in a peer-reviewed journal.

### Recruitment progress

Recruitment for the ORBITA-CTO pilot study commenced in October 2021. We have enrolled a total of 18 patients and randomised 13. Two further sites are planned to begin recruitment imminently.

### Optimisation of blinding for placebo and CTO PCI

The success of any double-blind, placebo-controlled trial rests on the robustness of the blinding of participants and assessors alike. There are many challenges to a randomised placebo-controlled trial in all complex high-risk interventions, including the CTO population. During ORBITA (11) and ORBITA-2 (12), the duration of PCI varied between 15 and 30 minutes allowing a manageable 15 minutes of extended procedure time for the placebo procedure. However, with CTO PCI, a procedure can last up to 2–3 hours (6) adding a further level of complexity to blinding. Therefore, masking participants' responses to temporal cues is a challenging but surmountable hurdle. There is further added complexity with CTO PCI in that, in routine clinical practice, most patients stay in the hospital overnight following the procedure. Here, we highlight the strategies used to blind participants effectively and to ensure that the blinding is as robust as possible based on our previous experience and a published framework for placebo-controlled trials of surgical procedures (18).

#### Preparation prior to catheterisation laboratory

Prior to the participant arriving at the day-case/admissions unit, all clocks and watches will be removed from the ward, the catheterisation laboratory, and connecting corridors. All digital clocks on observation screens will be concealed. Participants will switch off and store away their phones/digital devices. Participants will be placed in a side-room to avoid temporal unblinding by conversing with other patients. Participants will have a period of at least 60 minutes undergoing pre-procedural preparation on the ward prior to entering the catheterisation laboratory. Procedural start times will be staggered where possible in order to maintain the blinding of overnight ward staff. The CTO PCI operators will plan the procedural strategy prior to randomisation.

#### Procedure

In the catheterisation laboratory, equipment will be set up as is standard for CTO PCI including two procedural trolleys. Sensory deprivation techniques will be used to ensure blinding. Participants will wear over-the-ear headphones playing participant choice of music throughout the procedure to provide auditory isolation. All participants will have dual arterial access followed by dual injection coronary angiography as described above. Prior to randomisation, participants will be provided with further intravenous sedation and opiates to maintain a deep level of conscious sedation throughout the procedure. The blinded research fellow will then depart from the catheterisation laboratory and will not return to the catheterisation laboratories or the ward for a period of 6 hours.

Those allocated to the placebo group will undergo 60 min of simulated PCI by the catheterisation laboratory team. This will include gentle manipulation of the arterial sheaths, manoeuvring of the C-arm, inflation and deflation of indeflator as well as calling aloud requests for equipment to replicate a PCI procedure. All participants will receive one dose of IV Adenosine 140 μg/kg/min over 2 min prior to completion of the procedure to measure intracoronary physiology (including resting and hyperaemic indices, bolus and continuous thermodilution-derived measures of flow and resistance) in the PCI participants or to simulate symptoms with adenosine in the placebo participants, to maintain blinding in both groups.

In order to maintain participant blinding, no clocks or devices will be present for 2 h post-procedure. This will remove the potential for temporal cues for the participant once they leave the catheterisation laboratory and are admitted to a cardiology ward bed.

#### Staff blinding

Catheterisation laboratory staff, including operators, will not have any contact with participants after the conclusion of the procedure. To avoid day-case unit and ward staff becoming unblinded due to awareness of procedure start times, the laboratory staff will convey the patient to the laboratory and then directly to the bed on the overnight stay ward with a protocolised handover.

A standardised documentation of procedure will be safely stored separately and accessible in case of emergency or need for unblinding. A scripted handover to ward staff and template discharge paperwork to the GP and participant will be provided. All participants will be treated as if they had CTO PCI, with documentation clearly stating participants have undergone blinded randomisation.

All these methods (Table 2) to optimise placebo procedure and blinding are based on the DITTO framework (19) that has been proposed as a mechanism to optimise the design of placebo-controlled studies in randomised controlled trials.

**Table 2:** Optimisation of placebo procedure and blinding using DITTO framework.

Components	CTO PCI	Placebo intervention
Sedation	IV sedation + - Fentanyl	IV sedation + - Fentanyl
Before access	Skin preparation, positioning, draping, attachment of monitoring equipment	Skin preparation, positioning, draping, attachment of monitoring equipment
Anaesthesia	Local anaesthetic	Local anaesthetic
Access	Bilateral 7 Fr arterial access. Can be radial or femoral.	Bilateral 7 Fr arterial access. Can be radial or femoral.
Coronary angiogram	Bilateral coronary angiograms	Bilateral coronary angiograms
Radiation	Minimal number of views to delineate relevant coronary and CTO anatomy	Minimal number of views to delineate relevant coronary and CTO anatomy
Contrast	Minimal volume of contrast to delineate relevant coronary and CTO anatomy	Minimal volume of contrast to delineate relevant coronary and CTO anatomy
Guide catheter insertion	Guide catheter insertion. Operator determines best CTO PCI technique to achieve successful outcome.	N/A
Coronary CTO wiring, adjunctive device and balloon inflations and stent deployment	Coronary CTO technique including wiring, use of adjunctive devices, and DES implantation as per operator discretion to achieve best CTO PCI outcome.	N/A
Invasive and non-invasive physiological measurements	Measurements using dedicated catheters and wires. Administration of IV adenosine for hyperaemia indices.	IV infusion of Adenosine 140 μg/kg/min for 2 min prior to end of procedure to generate symptoms of chest tightness
Radiation	Minimal number of views, fluoroscopy, and cine runs to achieve successful CTO PCI	N/A
Contrast	Minimal volume of contrast to achieve successful CTO PCI	N/A
Closure of access	Removal of sheaths. TR band application if radial. Angioseal/FemoStop/Manual pressure for femoral access.	Removal of sheaths. TR band application if radial. Angioseal/FemoStop/Manual pressure for femoral access.
After access site closure	Apply dressing (optional)	Apply dressing (optional)
**Placebo optimisation strategies**		
Visual masking	Vertical screen.	
Auditory masking	Headphones playing participants choice of music applied once in catheter laboratory until completion of procedure. Removed prior to transfer to recovery.	
Physical cues	IV adenosine administration 140 μg/kg/min for 2 min in both PCI (as part of physiological assessments) and placebo groups.	
Time masking	Participants to have no access to clocks/time/devices once attends to day-case unit (or equivalent). Participant will have access to time 2 h post-procedure.	
Restriction of interaction between blinded/unblinded trial persons	Operators, catheter laboratory personnel will not interact with participant after procedure. Recovery and ward team will be blinded to randomisation allocation. Nursing handover will state participant to be treated as if they have had CTO PCI as part of research trial.	
Omission of intervention details in patient notes	Standardised procedural reports will be provided to maintain blinding. Handwritten procedural report kept in site file/online secure site only to be accessed in an emergency.	
Communication with GP	Standardised discharge summary including contact details for trial team to be provided for GP to maintain blinding.	

## Discussion

The ORBITA-CTO pilot study will provide a long-awaited assessment of the feasibility of a placebo-controlled study of CTO PCI and demonstrate the efficacy of using a novel, participant-tailored daily angina symptom app. There are multiple challenges to a placebo-controlled study of CTO PCI; however, this study has been designed to have robust and reproducible scientific methodology while trying to maintain a pragmatic approach to what is a complex cohort of chronic coronary syndrome patients.

One challenge in identifying patients who may potentially benefit from CTO PCI is establishing the presence of appropriate symptoms. CTO patients present with a variety of symptoms, including classical exertional angina and shortness of breath. Within the OPEN CTO registry, 19% (190 patients) reported only dyspnoea (20) as their main symptom, and therefore within ORBITA-CTO we will ensure that we capture this cohort (provided no alternative explanatory cause for breathlessness is identified at the multi-disciplinary team). Furthermore, the impact of collaterals combined with the related concept of warm-up angina (21) means CTO patients can have symptoms with variable intensity of exertion. With this in mind, for ORBITA-CTO, the symptom application will capture patient-specific symptoms and will also test if angina occurs with two provocative exercises of different intensities that will be pre-specified by the participant at enrolment. This avoids participants being labelled asymptomatic when, in reality, they may have adjusted their daily activities to prevent angina. We are aware that the theoretical higher fidelity for symptom assessment of the smartphone application is still to be realised in practise and will be demonstrated when the ORBITA-2 (12) study is published. We have therefore also included more conventional methods of symptom assessment, including the SAQ, the Rose dyspnoea scale, CCS, and the NYHA score.

CTO PCI success is associated with operator and hospital experience (22, 23). Therefore, the ORBITA-CTO Pilot study will be conducted at experienced CTO PCI centres (defined as those with dedicated CTO operators, equipment, catheter laboratory slots, and MDT meetings, although the latter is not mandatory) with established expert CTO operators (defined as experts in all recanalization techniques with a minimum of 5 years' experience and a volume of 40 CTO PCI's per year). This, combined with the inclusion severity of J-CTO ≤3 (a J-CTO score of 3 would be classified as “very difficult”), will allow maximal CTO PCI success in the study. J-CTO 4 and 5 category lesions are associated with reduced success rates (14, 24), and therefore by excluding these cases we aim to reduce the likelihood of failed cases within the PCI arm. However, the complexity included will still encompass the majority of CTO PCI offered at most centres. Importantly, randomisation occurs prior to any procedure being undertaken, so allocation will not be influenced by procedural success.

The ORBITA-CTO pilot study takes a pragmatic approach to anti-anginal medication optimisation. Initially, all participants must demonstrate symptoms while on optimal anti-anginal medications at baseline. We will ensure that participants are on two anti-anginal medications as a minimum to improve registry data, in which 38% of patients were on two anti-anginals, and 16.1% on 3 or 4 (25). Following randomisation procedure, these medications will be stopped, which allows a true comparison of CTO PCI vs. placebo. Participants will be informed that symptoms post CTO PCI are not unexpected as residual angina is reported in 20% of patients (25). Subsequently, anti-anginal medications will be re-introduced when participants contact the blinded research fellow, informing them of significant angina. This is reflective of real-life situations, where patients' post-procedure would report to their primary care physician if they had ongoing or recurrence of symptoms.

### Limitations

The lack of anatomical or ischaemic metrics at follow-up may be considered a limitation. However, our study has been designed to be pragmatic by evaluating participants for the primary indication of symptoms. Reduction in patient symptoms is the primary clinical goal of CTO PCI, regardless of ischaemia or residual stenosis. Some participants may remain or become symptomatic due to residual or acquired stenosis during the course of CTO PCI (side-branch ostial occlusion or loss); this too reflects real-world patient experience and is important to measure.

Although the study is not powered to identify efficacy, we have considered the fidelity of the angina ordinal clinical outcome scale alongside the design of the study as there may be a signal for efficacy with the study cohort. Furthermore, this study will contribute to the statistical power of a pivotal study.

### Impact on daily practice

CTO PCI has mixed results for symptomatic benefit. A randomised placebo-controlled study is required to evaluate this pertinent question given the significant resource utilisation, procedural risk, and financial cost of CTO PCI. This study will assess the feasibility of such a study in this cohort and evaluate any signal of symptomatic impact of CTO PCI.

## Ethics statement

The study involves human participants and was reviewed and approved by the Bradford Leeds Research Ethics Committee (21/YH/0165), United Kingdom. The patients/participants provided their written informed consent to participate in this study.

## Author contributions

SK, CC, POK, EB, PK, KT, GK, TK, RA-L, and JD contributed to conception and design of the study. SK, CR, SF, RS, KT, SM, PK, POK, SG, JS, KT, and JD contributed to acquisition of the data. SK wrote the first draft. RA-L, EB, JS, GK, TK, and JD wrote sections of the manuscript. All authors contributed to the article and approved the submitted version.

## APPENDIX

### APPENDIX A: Exclusion criteria

1. Acute coronary syndrome within 4 weeks.
2. PCI to non-CTO lesion in prior 4 weeks as part of acute coronary syndrome (ACS) or elective PCI.
3. Non-revascularised clinically important non-CTO vessel.
4. Proven ischaemia (invasive or non-invasive) in non-culprit territory.
5. Contraindications to PCI or drug-eluting stent (DES) implantation.
6. Inability to tolerate or contraindication to DAPT.
7. Severe valvular heart disease.
8. Severe chronic pulmonary disease (forced expiratory volume (FEV1) <30% of predicted value).
9. Severe musculoskeletal disease resulting in immobility.
10. Life expectancy <2 years.
11. Pregnancy.
12. Age <18 years.
13. Inability to consent.

### Appendix B: Anti-anginal uptitration

The preferred sequence of anti-anginal uptitration will be as follows:

Bisoprolol, Nifedipine MR or Amlodipine, Isosorbide mononitrate, Nicorandil, Ranolazine, and Ivabradine.

#### Units of anti-anginal

The following doses will be considered one “unit” of antianginal medication:

Bisoprolol 5 mg OD, amlodipine 2.5 mg OD, nifedipine MR 10 mg BD, isosorbide mononitrate MR 30 mg OD, isosorbide mononitrate SR 25 mg, ranolazine 375 mg BD, nicorandil 10 mg BD, and ivabradine 5 mg OD
